# Distinct Extracellular RNA Profiles in Different Plasma Components

**DOI:** 10.3389/fgene.2021.564780

**Published:** 2021-06-21

**Authors:** Jing Jia, Shangdong Yang, Jinyong Huang, Hong Zheng, Ying He, Liang Wang

**Affiliations:** ^1^Department of Medical Genetics & Cell Biology, School of Basic Medical Sciences, Zhengzhou University, Zhengzhou, China; ^2^Department of Tumor Biology, H. Lee Moffitt Cancer Center and Research Institute, Tampa, FL, United States; ^3^Department of Pathology, Medical College of Wisconsin, Milwaukee, WI, United States

**Keywords:** exRNA, plasma, ligation-free, RNA-seq, liquid biopsy

## Abstract

Circulating extracellular RNAs (exRNAs) have great potential to serve as biomarkers for a wide range of diagnostic, therapeutic, and prognostic applications. So far, knowledge of the difference among different sources of exRNAs is limited. To address this issue, we performed a sequential physical and biochemical precipitation to collect four fractions (platelets and cell debris, the thrombin-induced precipitates, extracellular vesicles, and supernatant) from each of 10 plasma samples. From total RNAs of the 40 fractions, we prepared ligation-free libraries to profile full spectrum of all RNA species, without size selection and rRNA reduction. Due to complicated RNA composition in these libraries, we utilized a successive stepwise alignment strategy to map the RNA sequences to different RNA categories, including miRNAs, piwi-interacting RNAs, tRNAs, rRNAs, lincRNAs, snoRNAs, snRNAs, other ncRNAs, protein coding RNAs, and circRNAs. Our data showed that each plasma fraction had its own unique distribution of RNA species. Hierarchical cluster analyses using transcript abundance demonstrated similarities in the same plasma fraction and significant differences between different fractions. In addition, we observed various unique transcripts, and novel predicted miRNAs among these plasma fractions. These results demonstrate that the distribution of RNA species and functional RNA transcripts is plasma fraction-dependent. Appropriate plasma preparation and thorough inspection of different plasma fractions are necessary for an exRNA-based biomarker study.

## Introduction

Extracellular RNA (exRNA) profiling is a promising tool to recapitulate gene expression changes that result from disease-specific alterations. Previous studies have shown that exRNAs may be used as biomarkers for monitoring dynamic changes of disease status (Soekmadji et al., 2018; Das et al., 2019; Zhou et al., 2019) and for cancer detection, recurrence evaluation, and survival prediction (Yuan et al., 2016; Max et al., 2018; Shah et al., 2018). However, exRNAs have a wide variety of classification and distinct size distributions. The major classes of exRNAs includes RNAs encapsulated in extracellular vesicles (EVs), RNAsoutside EVs but associated with various kinds of RNA-binding proteins, and true “cell-free” RNAs. These exRNAs are also present in cell debris, platelets, and nucleosomes (Joosse and Pantel, 2015; Snyder et al., 2016). Recent RNA-seq studies have shown a full spectrum of exRNA transcripts. In addition to evidence of small non-coding RNAs, such as miRNAs or piRNAs, there is increasing evidence supporting the presence of other RNA types, including lncRNAs, circRNAs, protein-coding RNAs, rRNAs, and tRNAs in plasma as well as in EVs (Chiou et al., 2018; Fanale et al., 2018; Li et al., 2018a). Therefore, it is necessary to inspect RNA abundance and diversity. Characterizing exRNA profiles in body fluids will facilitate an understanding of their origination and will pave the way toward more targeted biomarker discoveries.

Plasma is commonly used in clinical practice. Due to its complex compositions, plasma is also commonly used for biomarker discovery. Plasma is isolated from blood centrifuged at low speed. Although most red blood cells and mononuclear cells are effectively removed, a significant amount of cell debris and platelets remain after centrifugation. To isolate EVs in plasma, differential ultracentrifugation has been adopted (Thery et al., 2006). This density gradient centrifugation has also been used to isolate and characterize EV subpopulations, such as large and small EVs (Vagner et al., 2018). To simplify EV isolation, polyethylene glycol (PEG) has been used to promote EV precipitation (Rider et al., 2016). However, other RNA carriers in plasma could also be co-precipitated during the process (Karttunen et al., 2019). In addition, plasma treated by thrombin contains a high abundance of fibrin, which can be precipitated along with its binding RNAs by simple centrifugation. Although RNA profiles from EVs and whole plasma samples have been reported (Arroyo et al., 2011; Turchinovich et al., 2011; Allen et al., 2018; Driedonks et al., 2020), the comprehensive analysis of RNA profiles among different plasma fractions is needed. Furthermore, due to low yield and significant degradation of exRNAs, plasma-derived RNA profiling analysis is technically challenging. Most previous studies focused on either small or long RNA transcripts by using standard adaptor ligation and size selection during library preparation (Buschmann et al., 2016). Both adaptor ligation and size selection are known to have significant bias toward the enrichment of specific RNA types. Therefore, a comprehensive analysis of RNA profiles in different plasma fractions using less biased library preparation method is urgently needed.

In this study, we used a ligation-free RNA library preparation method and directly performed a sequencing analysis of the entire library, without size selection. We evaluated exRNA profile differences and identified unique features among different plasma fractions. We found that each plasma fraction had its own unique RNA composition as well as distribution of RNA species. The enrichment of specific RNA molecules or RNA species further emphasizes importance of quantifying full spectrum RNAs in each plasma fraction, since low abundance of biomarkers are often masked by the other components in the plasma (Boukouris and Mathivanan, 2015). The proper fractionation of plasma may facilitate discovery of the bona fide biomarkers that can be translated into clinical applications. Our study demonstrates that thorough inspection of all plasma fractions is necessary for exRNA-based biomarker study.

## Materials and Methods

### Plasma Collection

The objective of this study was to explore the potential differences of the RNA profiles in different plasma fractions. To accomplish this goal, 10 plasma samples (Table 1) were collected from the Medical College of Wisconsin tissue bank. After blood draw (EDTA anticoagulant), plasma was isolated by 2,000 *g* for 10 min at room temperature and stored at –80°C until use. The use of the human biospecimens was approved by the Institutional Review Board of the Medical College of Wisconsin and H. Lee Moffitt Cancer Center and Research Institute. Hematology analyses were performed to test the plasma component (Hematology analyzer, scil Vet ABC).

**TABLE 1:** Patient information and clinical diagnosis.

Sample ID	Age	Gender	Disease status
1	48	Male	Lung cancer
2	56	Male	Lung cancer
3	58	Female	Lung cancer
4	65	Male	Lung cancer
5	47	Female	Lung cancer
6	73	Male	Tongue cancer, secondary lung cancer
7	67	Male	Prostate cancer
8	72	Male	Prostate cancer
9	83	Male	Colon cancer
10	61	Female	Colon cancer

### Plasma Fractionation and RNA Isolation

Two hundred microliter of plasma of each sample was thawed from −80 to 4°C, and 100 U of RNA inhibitor (SUPERase⋅ In; Thermo Fisher Scientific, Waltham, MA, United States) was added to the plasma sample before centrifugation. Samples were first centrifuged at 500 *g* at 4°C for 10 min to precipitate cell debris and platelets. The supernatant was transferred to a new tube, and the leftover pellet (platelets and cell debris fraction or the Precipitate 1) was mixed with QIAzol lysis buffer (miRNeasy Micro Kit, QIAGEN, Germantown, MD, United States) for RNA extraction. 1/100 volume of thrombin (Thrombin Plasma Prep, SBI, Palo Alto, CA, United States) was added to the supernatant, which was then incubated at room temperature for 5 min and spun at 11,000*g* for 5 min at 4°C. After transferring the supernatant to a new tube, the pellet (the thrombin-treated fraction or the Precipitate 2) was mixed with QIAzol lysis buffer for RNA extraction. The supernatant was then mixed with 1/4 volume of Exoquick reagent (SBI), incubated at 4°C for 1 h, and then centrifuged at 1,500 *g* at 4°C for 30 min. After aliquoting the supernatant to a new tube, the pellet was suspended in 100 μl 1× phosphate buffer saline. QIAzol lysis buffer was added to the suspended pellet fraction (EV Precipitate) and a final supernatant fraction (leftover), separately. RNA isolation was performed as per the manufacturer’s standard protocol. DNase I was used to remove any residue DNA on the column before RNA elution. The extracted RNA was eluted in 14 μl of RNase-free water and stored at −80°C until use.

### Characterization of Plasma Fractions Using LC-MS/MS

A nanoflow ultra-high performance liquid chromatograph (RSLC, Dionex, Sunnyvale, CA, United States) coupled to an electrospray bench top orbitrap mass spectrometer (Q-Exactive plus, Thermo Fisher Scientific, San Jose, CA, United States) was used for tandem mass spectrometry peptide sequencing experiments following a standard protocol. MaxQuant software was used to identify and quantify the proteins. This work was conducted by the Proteomics and Metabolomics Core Facility at the H. Lee Moffitt Cancer Center & Research Institute.

### RNA Library Preparation and Sequencing

Library preparation was performed using the CATS (Capture and Amplification by Tailing and Switching) kit (Diagenode, Belgium) per manufacturer’s instructions. The ligation-free library preparation kit has been reported to reduce ligation bias and adaptor dimers (Dard-Dascot et al., 2018). Eight microliter of RNA (without fragmentation) was first subjected to RNA dephosphorylation and tailing. Subsequently, cDNA strand synthesis was performed with the anchored poly(dT) oligonucleotide containing a 3′-adaptor sequence. Next, the template-switching oligonucleotide was added to the cDNA product. The second cDNA strand was generated during the first cycle of the standard PCR reaction. A total of 15 amplification cycles were performed to ensure efficient library yield and to avoid the excessive over-amplification product at the same time. The final amplified libraries were purified by AMPure XP beads (Beckman Coulter). Qubit fluorometer was used to determine the concentration of the final libraries. All sequencing was done using an Illumina Sequencing instrument (50 bp single-end reads).

### Sequencing Data Analysis

Initial quality check of the sequencing data (FASTQ files) was done with FastQC (version 0.11.9, Babraham Bioinformatics). Cutadapt (version 2.3.1) was used to trim the FASTQ files for removal of 5′-GGG, 3′ Poly(A) tails and 3′-adaptor sequences. Sequences of <16 bases were also discarded. DNASTAR Lasergene 15.3 (Madison, WI, United States) was used for mapping. DESeq2 R package (version 1.26.0) was applied for data normalization.

To comprehensively evaluate RNA profiles, we adopted a stepwise alignment strategy. For each new RNA species, unmapped sequence reads from the previous step were used. The order of mapping was miRNAs, piRNAs, tRNAs, ncRNAs, protein-coding RNAs, circRNAs, rRNAs, and genome reference. The miRNA databases (release 22.1) were downloaded from miRBase, piRNAs from piRNA Quest, tRNAs from gtRNAdb, and circRNAs from cirBase. The lincRNAs, snoRNAs, and snRNAs came from the GRCh38 ncRNA release 96. For protein-coding mRNA and other RNA types, sequence reads were mapped to the Gencode version 24. rRNA mapping was based on both of the GRCh38 ncRNA and 45S, 5S, and mt-rRNA sequences.

Prior to comparative analysis among different fractions, we performed sample-level quality control using Principal Component Analysis (PCA). Samples with <1 million mapped reads were excluded. Outliers identified by the PCA were excluded from further analysis. Overall clustering and distribution of categorized RNA profiles through t-SNE plots were done by using Rtsne package (version 0.15). Heatmap visualization was generated by pheatmap package (version 1.0.12). Gene ontology (GO) analysis was performed with clusterProfiler package (version 3.14.3). After mapping to known RNA databases, unmapped reads were used for novel miRNA prediction by miRDeep2 package (version 2.0.1.2). The newly identified RNAs were validated by comparing to a small RNA sequencing database (DASHR, or database of small human non-coding RNAs) v2.0 (Kuksa et al., 2019).

### Statistical Analyses

Statistical analyses of the fraction-specific transcript differences were performed using one-way ANOVA. If the data failed the Levene’s test for homogeneity of variances, Kruskal-Wallis test was applied. A *p* value <0.05 was considered significant. To correct for multiple testing, false discovery rate (FDR) was applied. FDR < 0.05 was defined as statistical significance.

## Results

### Characteristics of Four Plasma Fractions

**FIGURE 1:**
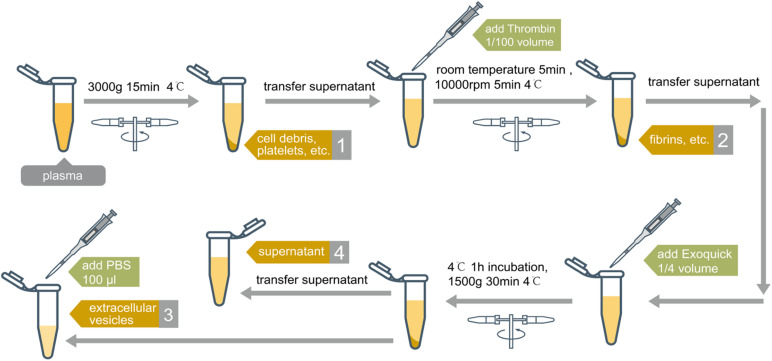
Separation process of plasma samples. Four fractions were obtained after sequential physical and chemical precipitation: (1) platelets and cell debris, named precipitate 1; (2) fibrins, named precipitate 2; (3) extracellular vesicles, named EV precipitate; (4) supernatant, named leftover fraction. Except for supernatant, each fraction was suspended by 100 μl of PBS Buffer before RNA extraction.

To evaluate exRNA profiles in different plasma fractions, we performed sequential physical precipitation (centrifugation) and chemical precipitation (Thrombin and Exoquick exosome precipitation solution treatment) (Figure 1). The Precipitate 1was a pellet by centrifugation of the platelet-rich plasma samples. This fraction enriches cell debris, platelets, and apoptotic bodies. After treating the supernatant with thrombin followed by centrifugation, the Precipitate 2 was collected. This fraction is mainly comprised of the precipitated fibrins. The EV Precipitate is obtained by centrifugation of Exoquick-treated and fibrin-depleted plasma (Taylor et al., 2011). The leftover fraction is the final leftover supernatant after the three rounds of centrifugation.

To characterize different plasma fractions, we performed LC-MS/MS to detect and quantify protein molecules in each fraction. This analysis showed that thrombospondins, matricellular proteins, were enriched in Precipitate 1. Fibrinogen alpha and gamma chains were enriched in Precipitate 2. Tetraspanins (CD9), most often used as specific exosome markers, were detected in EV Precipitate only. RNA binding proteins (Annexin A2 and trafficking/sorting associated proteins RAB1B) were also enriched in EV Precipitate while Lipoprotein particles such as ApoAI and ApoB100 were highly abundant in all plasma fractions (Table 2 and Supplementary Table 1). To confirm the existence of platelets, we performed a hematology analysis (Hematology analyzer, scil Vet ABC) of the plasma samples. This analysis showed 94 platelets 10^3^/mm^3^ in the initial plasma samples; the platelets were highly enriched in Precipitate 1 (391 PLT 10^3^/mm^3^) and significantly deprived in the supernatant after first centrifugation (6 PLT 10^3^/mm^3^) (Supplementary Table 2).

**TABLE 2:** Characterization of plasma fractions by LC-MS/MS.

Category	Protein names	Precipitate 1	Precipitate 2	EV Precipitate
Thrombospondin	TSP4	1.23E + 08	1.48E + 06	0
Fibrinogen	FIBA	1.70E + 11	5.83E + 11	1.10E + 11
Fibrinogen	FIBG	1.84E + 11	4.76E + 11	9.68E + 10
Tetraspanins	CD9	0	0	3.86E + 07
RNA binding proteins	ANXA2	0	0	2.32E + 07
Trafficking/sorting	RAB1B; RAB1A	0	0	9.09E + 06
Lipoprotein	APOB	5.19E + 11	4.02E + 10	1.15E + 11
Lipoprotein	APOA;LPAL2	1.24E + 09	7.06E + 08	3.12E + 09

### exRNA Size and Read Count Distribution in Different Plasma Fractions

**FIGURE 2:**
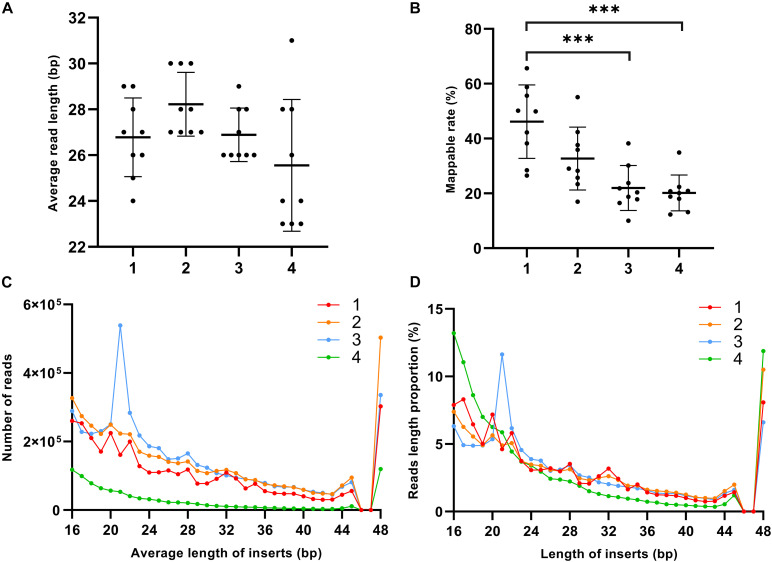
Distribution pattern of exRNA size and read count in different plasma fractions. **(A)** Average insert size of each fraction (ns-not significant, *p* > 0.05, one-way ANOVA). **(B)** Mappable rate of each fraction (*p < 0.05, **p < 0.01, ***p < 0.001, one-way ANOVA). **(C)** Read count of average length of inserts. **(D)** Proportion of size distribution. Solid line represents each fraction. The numbers 1–4 represent Precipitate 1, Precipitate 2, EV Precipitate, and the leftover, respectively.

For each fraction of the 10 plasma samples, we made a separate sequencing library for a total of 40 libraries, and screened all raw reads with MultiQC (Ewels et al., 2016). After the initial quality checking, one outlier sample from each fraction was filtered out from further analysis (sample 9 in Precipitate 1, sample 5 in the Precipitate 2 and EV Precipitate, and sample 3 in the leftover). Different fractions in the nine remaining plasma samples showed a dramatic difference in either insert size or mappable rate. Precipitate 2 had the longest insert size on average, and the leftover showed highly variable size (Figure 2A and Supplementary Table S3). We observed a gradual decrease of mappable reads from Precipitate 1 to leftover. On average, Precipitate 1 had a mappable rate of ∼46.2% while leftover showed a mappable rate of ∼20.2% (Figure 2B). Overall, smaller inserts tended to have higher read counts. EV Precipitate showed a sharp peak at 21 nt, which accounted for 11.6% of all mapped transcripts (Figures 2C,D). This was not a surprising finding because small miRNAs were enriched in the fraction (Zhang et al., 2017). Additionally, a suden decrease and then increase at 46–47 nt was observed because we trimmed 3 nt from the 50 bp sequencing length and combined all reads longer than 48 bp inserts. Although mappable reads were low (Figure 2C), leftover contained a significantly higher percentage of small (<20 bp) and large fragments (>47 bp) (Figure 2D). The sharp increase after 47 nt suggested the presence of a significant number of longer RNA transcripts in leftover.

### Overall exRNA Compositions in Plasma

To determine exRNA profiles, we mapped the sequencing data to a variety of reference RNA sequences using a stepwise mapping strategy and detected a total of 140,914 RNA transcripts across all samples. Of all detected transcripts, 10,618 passed the threshold, with read counts ≥10 in at least 1 sample (Table 3). To better illustrate the transcript spectrum of each fraction, we divided all transcripts into 11 categories based on the source of reference and the current popular consensus toward the exRNA classification (Yuan et al., 2016; Shah et al., 2018). To better evaluate various classes of RNA species, we adopted high stringent mapping parameters to each reference sequence (Supplementary Table 4). For some RNA categories, we detected most of the sequences in reference database. For example, we observed 2,465 unique miRNAs from a total of 2,656 known miRNAs in the miRNA database, and 436 unique tRNAs from a total of 442 tRNA references, including some special form of tRNAs such as selenocysteine tRNA (tRNA^SeC^). EV Precipitate had an average read count of 651.2 for tRNA: SeC-TCA-1-1—the highest among all four fractions. However, leftover fraction did not have any read count for this tRNA. The EV precipitate had the largest number of miRNA count and smallest deviation, which met our expectation. For piRNAs and circRNAs, the proportion of their transcripts to the total number of detected transcripts in the corresponding reference database was 14.0 and 9.2%, respectively (Supplementary Table 5).

**TABLE 3:** RNA categories and sources of RNA references for mapping.

Category	Composition	Source	No. of transcript	No. of transcript (read cutoff ≥10)
miRNAs	microRNA (both mature and premature)	miRBase Release 22.1 GRCh38 ncRNA release 96	2,465	359
piRNAs	Piwi-interacting RNA	piRNAQuest	1,568	331
tRNAs	tRNA, mt-tRNA	gtRNAdb GRCh38 ncRNA release 96	436 (22 of mt-tRNA)	358 (22 of mt-tRNA)
rRNAs	rRNA, mt-rRNA	45S, 5S, mt-rRNA sequences GRCh38 ncRNA release 96	65	51
lincRNAs	long intergenic non-coding RNA	GRCh38 ncRNA release 96	8,686	1,267
snoRNAs	small nucleolar RNA	GRCh38 ncRNA release 96	443	125
snRNAs	small nuclear RNA	GRCh38 ncRNA release 96	856	221
circRNAs	circular RNA	circBase	12,910	1,872
other ncRNAs	3prime overlapping ncRNA, asRNA, bidirectional promoter lncRNA, macro lncRNA, miscRNA, non-coding, processed transcript, retained intron, ribozyme, scaRNA, scRNA, sense intronic, sense overlapping, sRNA, TEC, vaultRNA	GRCh38 ncRNA release 96	10,838	1,959
protein-coding RNAs	protein-coding RNA	Gencode version 24	94,289	3,816
other RNAs	immunoglobulin, T cell Receptor, pseudogene	Gencode version 24	8,358	259
**Total**			140,914	10,618

### Distinctive Spectrum of exRNA Transcripts Among Different Plasma Fractions

**FIGURE 3:**
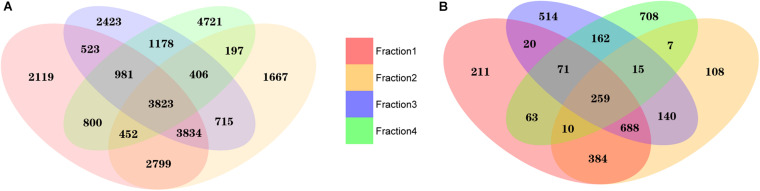
Unique and overlapping transcripts in four plasma fractions. **(A)** Average read count of transcripts ≥1 in each fraction. **(B)** Average read count of transcripts ≥10 in each fraction. The numbers 1–4 represent fractions for Precipitate 1, Precipitate 2, EV Precipitate, and the leftover, respectively.

To compare exRNA profiles, we first performed an intersection analysis. Although significant overlaps remained, we observed a distinct distribution of mapped exRNAs among different plasma fractions (Figure 3). When the average read count was ≥1 in each fraction, we detected a total of 15,331, 13,893, 13,883 and 12,558 transcripts in each of the fractions, respectively. Among these transcripts, 3,823 were common in all fractions. Furthermore, 2,119, 1,667, 2,423, and 47,21 transcripts were unique in Precipitate 1, Precipitate 2, EV Precipitate and leftover, respectively. When the average read count was ≥10, 1,706, 1,611, 1,869, and 1,295 transcripts were detectable in these corresponding fractions, respectively. Among those, 259 transcripts were shared in the four fractions, and 211, 108, 514, and 708 transcripts were unique in these fractions. Clearly, EVs Precipitate showed the largest number of detectable unique transcripts (read count ≥ 10) among all fractions, indicating RNA stability inside the vesicles. The leftover had the most unique transcripts among all the fractions.

**FIGURE 4:**
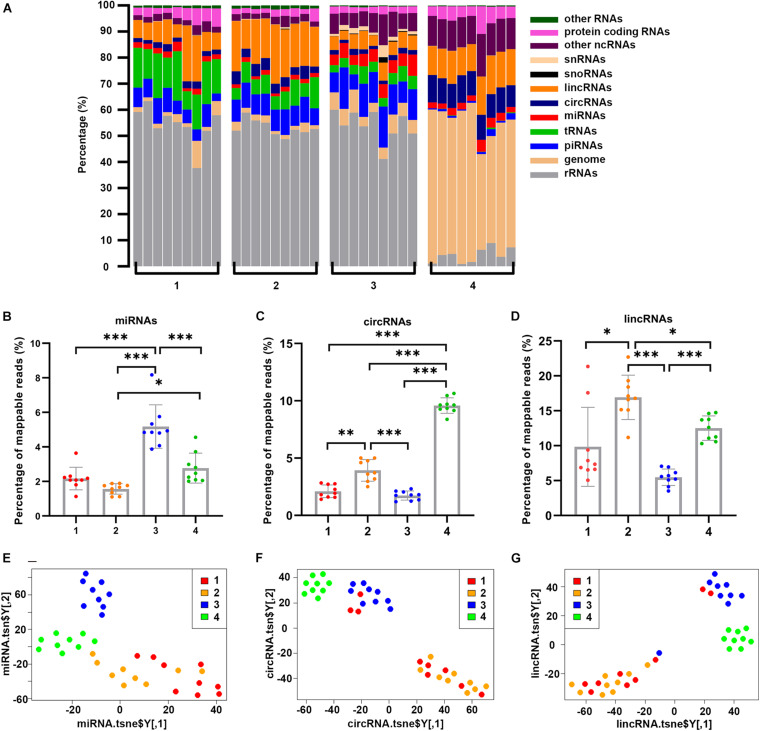
Distribution and clustering of RNA transcripts in all samples of each fraction. **(A)** Percentage of each RNA species in fractionated samples, grouped by four different fractions. The vertical bar represents each sample. **(B–D)** Percentage of read counts in four fractions for miRNAs, circRNAs, and lincRNAs datasets. Significant enrichment of miRNAs in the EV Precipitate, circRNAs in the leftover, and lincRNAs in both the Precipitate 2 and leftover was observed (*p < 0.05, **p < 0.01, ***p < 0.001, one-way ANOVA). **(E–G)** t-SNE plots for the 4 fractions based on genome, miRNAs, protein-coding transcript dataset. The numbers 1–4 represent fractions for Precipitate 1, Precipitate 2, EV Precipitate and the leftover, respectively.

We then examined the distribution of exRNA species in each fraction. The most noticeable difference among these fractions was rRNA species (Figure 4A). On average, rRNA accounted for 54.4, 53.1, and 54.1% of Precipitate 1, Precipitate 2 and EV Precipitate, respectively. However, rRNA represented only 4.4% in the leftover. In addition, compared to the leftover, the other 3 fractions contained significantly higher levels of tRNAs and piRNAs (0.2 vs. 8.4% and 1.1 vs. 8.8% on average). In fact, tRNA content showed a clear stepwise reduction from 13.8% in Precipitate 1 and 7.2% in the Precipitate 2 to 4.3% in the EV Precipitate and 0.2% in the leftover. Other ncRNAs (a category list in Table 1) showed a stepwise increase from 2.6% in Precipitate 1 to 12.2% in the leftover. Other noticeable differences included the significant enrichment of miRNAs in the EV Precipitate, circRNAs in the leftover, and lincRNAs in both the Precipitate 2 and leftover (Figures 4B–D). Interestingly, the leftover showed significantly higher reads (51.6%) mapped to the human genome for sequences that were not mapped to the known RNA references. A detailed comparison of RNA species between different fractions is shown in Supplementary Figure 1. Furthermore, t-distributed stochastic neighbor embedding (t-SNE) plots showed a clear separation between different fractions (Figures 4E–G). Regardless of RNA species, the leftover appeared to separate itself from the other 3 fractions, demonstrating its unique RNA characteristics (Supplementary Figure 2).

### Differentially Enriched Transcripts in Different Plasma Fractions

**FIGURE 5:**
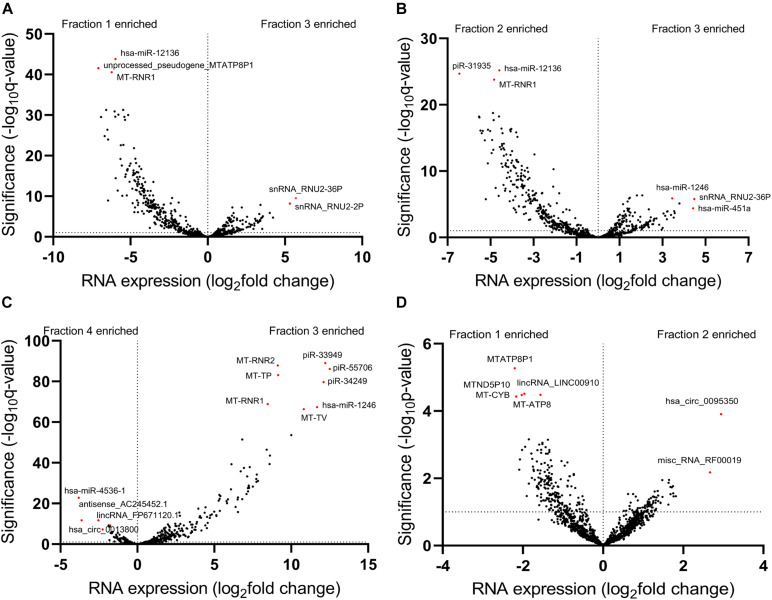
Differentially enriched transcripts between different fractions. **(A–C)** Volcano plots show differentially enriched transcripts of the EV Precipitate versus the other three fractions. **(D)** Volcano plot shows differentially enriched transcripts between Precipitate 1 and Precipitate 2. Horizontal dashed line represents the significance level of Benjamini-Hochberg FDR of 0.01 **(A–C)**, *p* < 0.05 **(D)**. The numbers 1–4 represent fractions for Precipitate 1, Precipitate 2, EV Precipitate and the leftover, respectively.

To further investigate exRNA profiles in each fraction, we performed a differential expression analysis by comparing transcript abundances from these fractions. We first compared the EV Precipitate to the other fractions and observed a total of 2287 differentially enriched transcripts in Precipitate 1 (FDR < 0.01), representing transcripts from all 11 categories (Supplementary Figure 3A). We also observed 1,922 and 4,056 differentially enriched transcripts in Precipitate 2 and leftover, respectively (Supplementary Figures 3B,C) (FDR ≤ 0.01). Interestingly, the EV Precipitate showed significantly more transcripts with lower abundance than those in Precipitate 1 and 2. Compared to the leftover, however, the EV Precipitate demonstrated more transcripts with higher abundance. Volcano plots of these differentially enriched transcripts showed that the EV Precipitate was likely to enrich miRNAs, snRNAs, and piRNAs. However, not all miRNAs were enriched in the EV Precipitate, miR-12136 was significantly higher in Precipitate 1 and 2, whereas miR-4536-1 was mainly present in the leftover (Figures 5A–C). Additionally, differences between Precipitate 1 and 2 were less obvious, and both fractions showed enrichment of mitochondria-related transcripts (Figures 5A,D).

**FIGURE 6:**
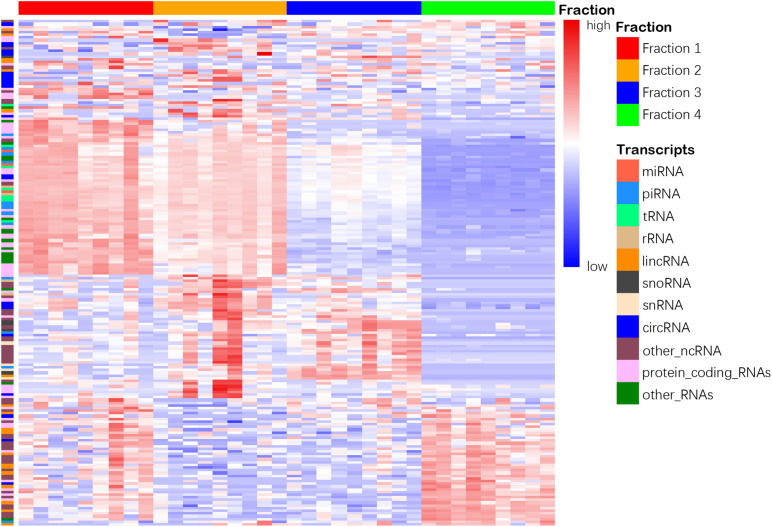
Heatmap shows distinct clustering of 4 plasma fractions. Top horizontal color bars represent 4 different fractions. One hundred eighty-seven differentially enriched transcripts in 11 RNA categories are shown in each row (adjusted *p* < 0.01). The numbers 1–4 represent fractions for Precipitate 1, Precipitate 2, EV Precipitate and the leftover, respectively.

We then compared the leftover to the other 2 fractions and found 4,596 and 4,985 differentially enriched transcripts in Precipitate 1 and 2, respectively (Supplementary Figures 3D,E). However, when compared Precipitate 1 to Precipitatec 2, only 187 transcripts showed differences in their abundance (Supplementary Figure 3F). Furthermore, we summarized the differentially enriched transcripts based on their RNA categories (Supplementary Table 6). Although the leftover had fewer enriched transcripts, compared to the other 3 fractions, the percentages of enriched lincRNAs were 45.2, 65.2, and 74.5%, respectively (Supplementary Figures 4A–C). A >2-fold increase was shown in 26 lincRNAs in the leftover when compared to the other three fractions. For snoRNAs and snRNAs, the EV Precipitate showed significant enrichment (Supplementary Figures 4D–I). Overall, the four fractions demonstrated distinct transcript profiles, which were defined by 187 differentially enriched transcripts (*p* < 0.01) (Figure 6).

### Novel miRNA Prediction

**FIGURE 7:**
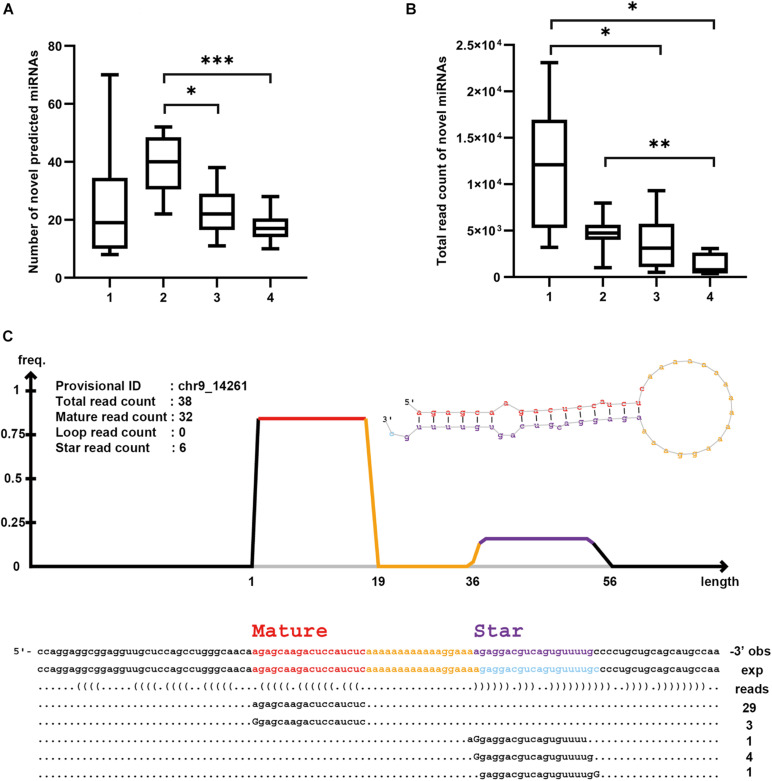
Novel miRNA prediction by miRDeep2. **(A)** Number of novel predicted miRNAs in each fraction (*p < 0.05, **p < 0.01, ***p < 0.001, one-way ANOVA). **(B)** Total read count of predicted novel miRNA in each fraction (*p < 0.05, **p < 0.01, ***p < 0.001, one-way ANOVA). **(C)** Novel miRNA on chr9_14261 (EV Precipitate, sample 10). Upper left shows the information about the sequences in the region. Upper right shows the secondary structure of the miRNA. Below are the mapped sequences on both star and mature strands. Number on the bottom right is read count of mapped sequences. Mismatched nucleotides are indicated by uppercase letters.

After mapping all sequence reads to known transcript databases, we examined the remaining reads to determine whether they contained any novel miRNAs. We applied miRDeep2 package and identified a total of 951 novel miRNAs, among which the Precipitate 2 showed the largest number of the predicted novel miRNAs (39.6 on average), whereas the Precipitate 1 had the largest number of total read count mapping to the novel miRNAs (11,707.9 on average) (Figures 7A,B). Interestingly, the EV Precipitate, which had the highest number of known miRNAs, showed much fewer novel miRNAs (23 on average) and related read counts (3,760.9 on average). These novel miRNAs were located in either the star or mature regions, and varied in length and in 3′/5′ end (Figure 7C). It is worth mentioning that some predicted miRNAs are fraction-dependent. For example, the novel miRNA at chr1: 630698–630779 (−) was found in all samples of Precipitate 1, but chr14:32485091-32485178 (+) was found in eight samples only. The novel miRNA at chr5:30930794-30930840(−) was observed in eight samples of the Precipitate 2 (Supplementary Table 7).

We performed *in silico* validation of these newly identified miRNAs using the DASHR 2.0 database and observed high-expression levels in these predicted miRNAs in the small RNA sequencing database (Kuksa et al., 2019). For example, predicted miRNA at Chr1: 630698-630779 (−) showed a significant peak with 10,995 reads per million. Representative novel miRNA loci with high small RNA read counts are shown in Supplementary Figure 5.

## Discussion

Plasma RNAs have been widely used for novel biomarker discovery. However, these previous studies use either whole plasma or a specific fraction of plasma, such as EVs (Huang et al., 2013; Jeppesen et al., 2019; Karttunen et al., 2019). So far, systematic examination of exRNA profiles among different plasma fractions and the knowledge of the RNA spectrum are limited. In this study, we applied a sequential centrifugation process to separate different plasma fractions. We performed ligation-free RNA-seq to profile the whole transcriptome in each fraction. Our data showed that different plasma fractions demonstrated a distinctive spectrum of exRNA transcripts; they not only varied in the library’s insert size but also differed in the enrichment of RNA species. Precipitate 1 is characterized by a large number of tRNAs and protein-coding RNAs, especially mitochondria-associated genes. Precipitate 2 is characterized by a large number of lincRNAs and protein coding RNAs, whereas small RNAs, such as miRNAs and piRNAs, are mainly enriched in the EV Precipitate. It is worth mentioning that these piRNAs require further validation since many non-coding RNA fragments may be falsely annotated as piRNAs (Tosar et al., 2018). Although the first 3 fractions contain most of the exRNAs, the leftover fraction had the highest percentage of circRNAs. This study provides evidence of exRNA profiling differences among different plasma fractions and emphasizes the importance of preanalytical plasma preparation in biomarker discovery.

It is well known that sample preparation methods including collection, storage, and isolation have a great effect on the subsequent data analysis. However, a consensus has not been reached on a uniform processing method (Gardiner et al., 2016). One study has shown that thrombin-triggered coagulation may trap EVs in the clot, leading to loss of EVs in precipitates (Arakelyan et al., 2016). In this study, we applied thrombin to remove fibrinogen and fibrin and, therefore, to increase the efficiency of EVs recovery. Our study showed that the fibrin-enriched fraction had its own unique RNA profile, characterized by the longest average insert length, a large proportion of lincRNAs, and enrichment in a platelet-related biological process. The lincRNAs are believed to participate in the coagulation-related functions, which are relevant to cellular stress response and cancer metastasis (Li et al., 2015; Leti and DiStefano, 2017). Thus, it is interesting to know the potential role of the lincRNA in the process of blood coagulation. Additionally, this result also suggests that the removal of highly abundant proteins is important before EV isolation in plasma. Meanwhile, exRNAs associated with or trapped by these RNA-binding proteins may also serve as novel markers for clinical use.

Our data showed that rRNAs accounted for most sequence reads in the first 3 fractions, whereas circRNAs were enriched in the leftover. Several studies have reported a predominance of rRNAs in EVs, which is consistent with our observation (Nolte-’t Hoen et al., 2012; Lefebvre et al., 2016; Sork et al., 2018). An increasing number of studies have highlighted the precise cleavage patterns of rRNA (Li et al., 2012; Fiskaa et al., 2016). These rRNA fragments have been shown to affect cell viability and mediate the RNA interference pathway, indicating a potential role in gene regulation (Weiberg et al., 2013; Hewson et al., 2016). However, the leftover contains significantly fewer rRNA species but other types of RNA, such as circRNAs (Thery et al., 2006). Due to exposure to RNases in the circulating system, non-protected linear RNAs are subjected to quick degradation. Therefore, the relative abundance of circRNAs in the leftover is significantly increased because of their stable structures. This unique feature helps capture an extensive spectrum of cirRNAs. However, circRNAs cannot be tailed due to their circular nature. Therefore, we expect that the captured circRNAs are partially degraded before library preparation. In addition, although the EV Precipitate is known to contain a higher percentage of small RNAs, including miRNAs and piRNAs, our data also showed enrichment other types of RNAs. For example, *MAGI1* is 4.9- and 6.1-fold higher in the EV Precipitate than in Precipitate 1 and 2, respectively. This gene is a member of the membrane-associated guanylate kinase homolog (MAGUK) family that participates in the assembly of complexes on the inner surface of the plasma membrane at regions of cell-cell contact, and also plays a role in tight junction (Laura et al., 2002).

Currently, most RNA-seq protocols require an adaptor ligation step, which is prone to ligation biases (Fuchs et al., 2015). Additionally, library size selection by gel cut or bead ratio may introduce additional biases. To address these issues, we adopted a less biased method, CATS (Diagenode). This RNA-seq method is ligation-free and is believed to capture the full spectrum of exRNA species from an extremely low input (Turchinovich et al., 2014).

Because miRNA biogenesis is a dynamic process, significant diversity has been reported in either the structure or splicing manner (Okamura et al., 2007). We found that several novel miRNAs were enriched in specific plasma fractions, suggesting the complexities of miRNA biogenesis and transportation. Meanwhile, miRNAs have been reported in mitochondrial mitoplasm. Their presence indicates the potential role of miRNAs in mitochondrial transcriptional regulation (Borralho et al., 2015). One example is a novel miRNA at chrM:5528-5609(−), which is a starting point of mitochondrial L-strand replication and transcription of 2 tRNAs. It would be interesting to know whether the novel miRNA is derived from the tRNAs (Li et al., 2018b). Our data showed that the predicted mitochondrial miRNA is highly enriched in Precipitate 1. Because the first fraction is characterized by high platelets counts, the mitochondrial-derived miRNA may also have an effect on the function of platelets. Further characterizationof the fraction-enriched miRNAs will not only assist in biomarker discovery but will also help understand the pathophysiological process of human diseases.

Despite our observation of distinct RNA profiles in different plasma fractions, this study has several limitations. First, we did not systematically characterize the exact components of each fraction. The separation of each fraction by centrifugation may produce precipitates that are composed of a wide variety of substances. A future study is needed to evaluate the quality of each fraction and to elucidate these substances and their RNA-binding preferences. Second, the sample size was small, and plasma samples were from patients with different cancer types. Further study using more clinical samples are needed to capture unique RNA signatures for these cancer types. Third, the stepwise alignment protocol may be biased to the mapping order of RNA species. The first RNA species used for mapping will have the advantage of using a full set of RNA-seq data. Finally, the current RNA-seq method has lower capacity to capture longer RNAs because of their more complicated secondary structure, low efficiency of reverse transcription, and PCR-based library amplification.

In summary, this study applied sequential centrifugation to collect 4 fractions of plasma samples and performed RNA-seq to examine the full spectrum of the exRNA transcriptome in these 4 plasma fractions. The study results show that fractionated plasma contains highly diverse exRNA species that cover all known RNA types in reference RNA databases. This study also shows that each plasma fraction has a unique distribution of these exRNA species, suggesting the complexity of using plasma for the biomarker discovery. Special plasma preparation may be needed when targeted exRNAs are fraction-specific. Because the different exRNA compositions may indicate specific functionality, this fractionated plasma analysis further extends our understanding of plasma’s role as a functional RNA delivery system.

## Data Availability Statement

The datasets presented in this study can be found in online repositories. The names of the repository/repositories and accession number(s) can be found below: https://www.ncbi.nlm.nih.gov/geo/, GSE145767.

## Ethics Statement

The studies involving human participants were reviewed and approved by Institutional Review Board of the Medical College of Wisconsin and Institutional Review Board of Moffitt Cancer Center. The patients/participants provided their written informed consent to participate in this study.

## Author Contributions

LW, JJ, and HZ conceived the presented idea. JJ and JH performed the experiments and primary data analysis. SY verified the analytical work. JJ wrote the manuscript with support from HZ, YH, and LW. LW supervised the project. All authors discussed the results and contributed to the final manuscript.

## Conflict of Interest

The authors declare that the research was conducted in the absence of any commercial or financial relationships that could be construed as a potential conflict of interest.
