# Supplementary material for: Distinct Extracellular RNA Profiles in Different Plasma Components
Source: Front Genet. 2021 Jun 21;12:564780. doi: 10.3389/fgene.2021.564780 (PMC8256274; doi:10.3389/fgene.2021.564780)
Supplement: Supplementary file 1 [file Data_Sheet_1.PDF]

**Supplementary material**

Figure S1. Comparisons of RNA species between different fractions.

Figure S2. Different types of RNA datasets showed different patterns of clustering by t-SNE plots.

Figure S3 Heatmap showed the clustering between each two of the fractions.

Figure S4. RNA category-specific clustering.

Figure S5. Representative novel miRNA loci in DASHR 2.0 database.

Table S1. Full list of protein components in three plasma fractions by LC-MS/MS analysis

Table S2. Hematology analysis of plasma samples before and after removal of precipitate 1.

Table S3. Average read length distribution.

Table S4. Mapping parameter settings to each reference sequence.

Table S5. Abundance of each RNA category.

Table S6. Differentially expressed transcripts between each fraction based on RNA category.

Table S7. miRDeep2_novel miRNAs prediction.

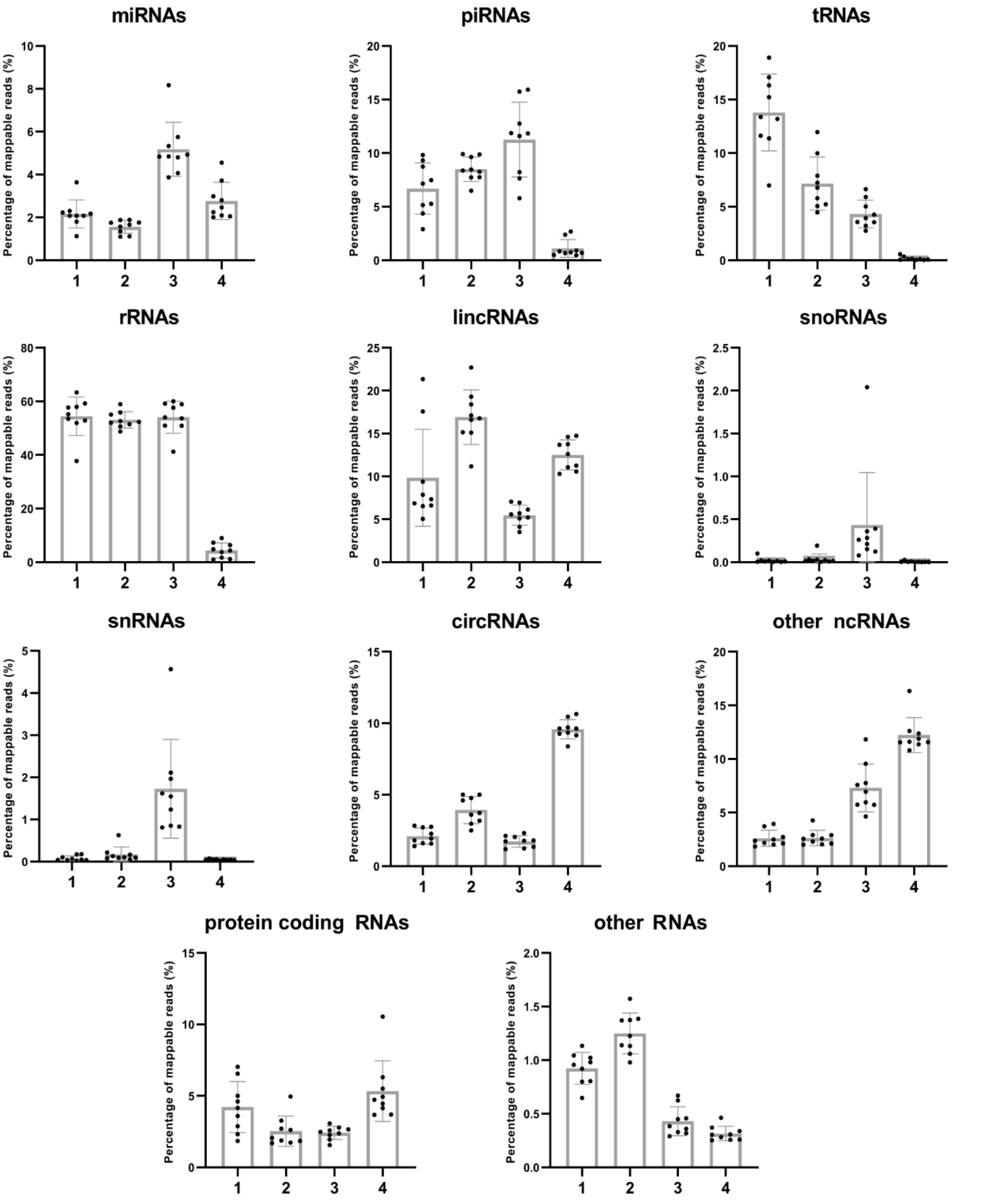


Figure S1. Comparisons of RNA species between different fractions. The numbers (1-4) on x axis represent precipitate 1, precipitate 2, EV precipitate and leftover fraction, respectively.

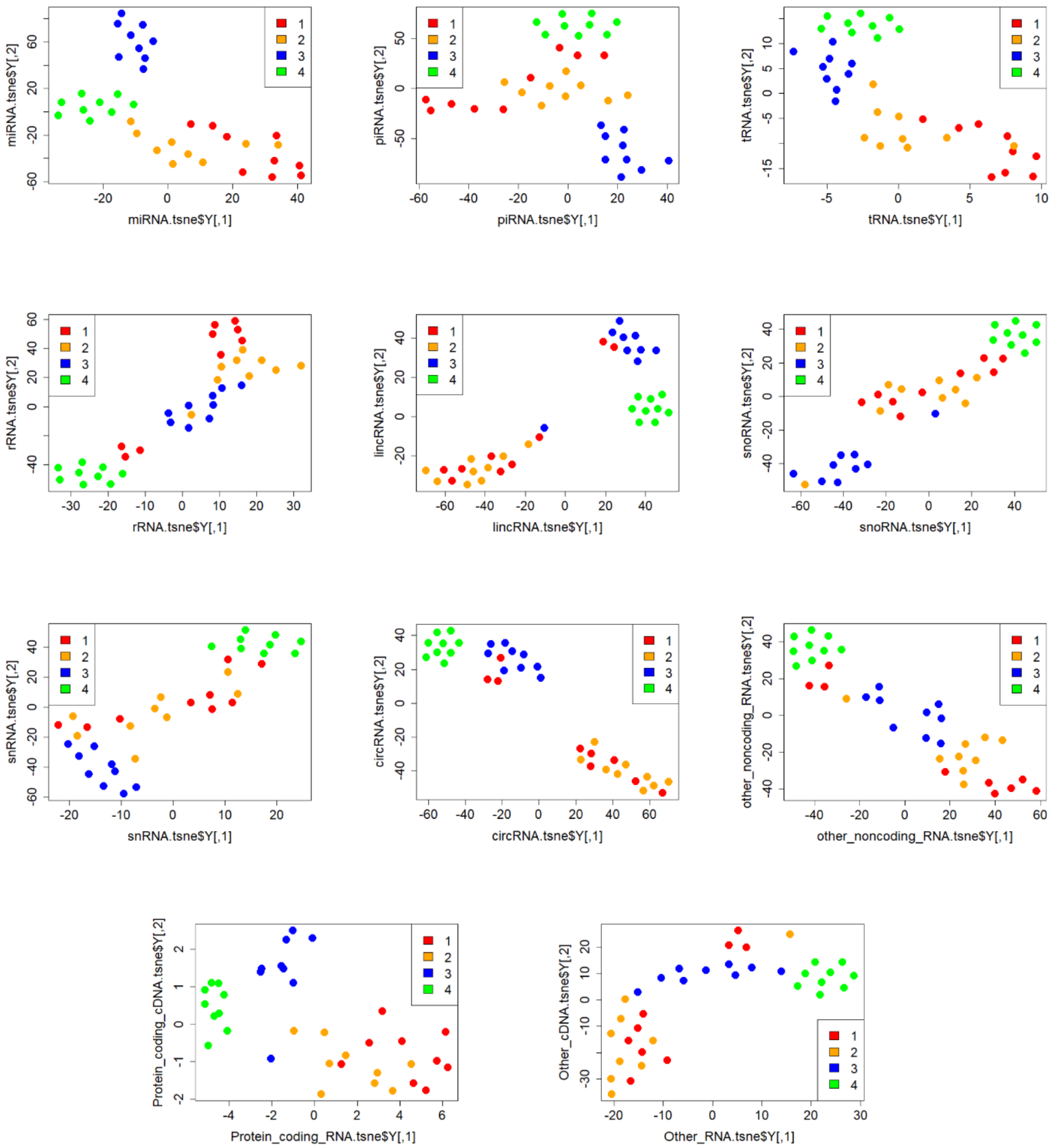


Figure S2. Different types of RNA datasets showed different patterns of clustering by t-SNE plots. The color dots and their corresponding numbers (1-4) represent precipitate 1, precipitate 2, EV precipitate and leftover fraction, respectively.

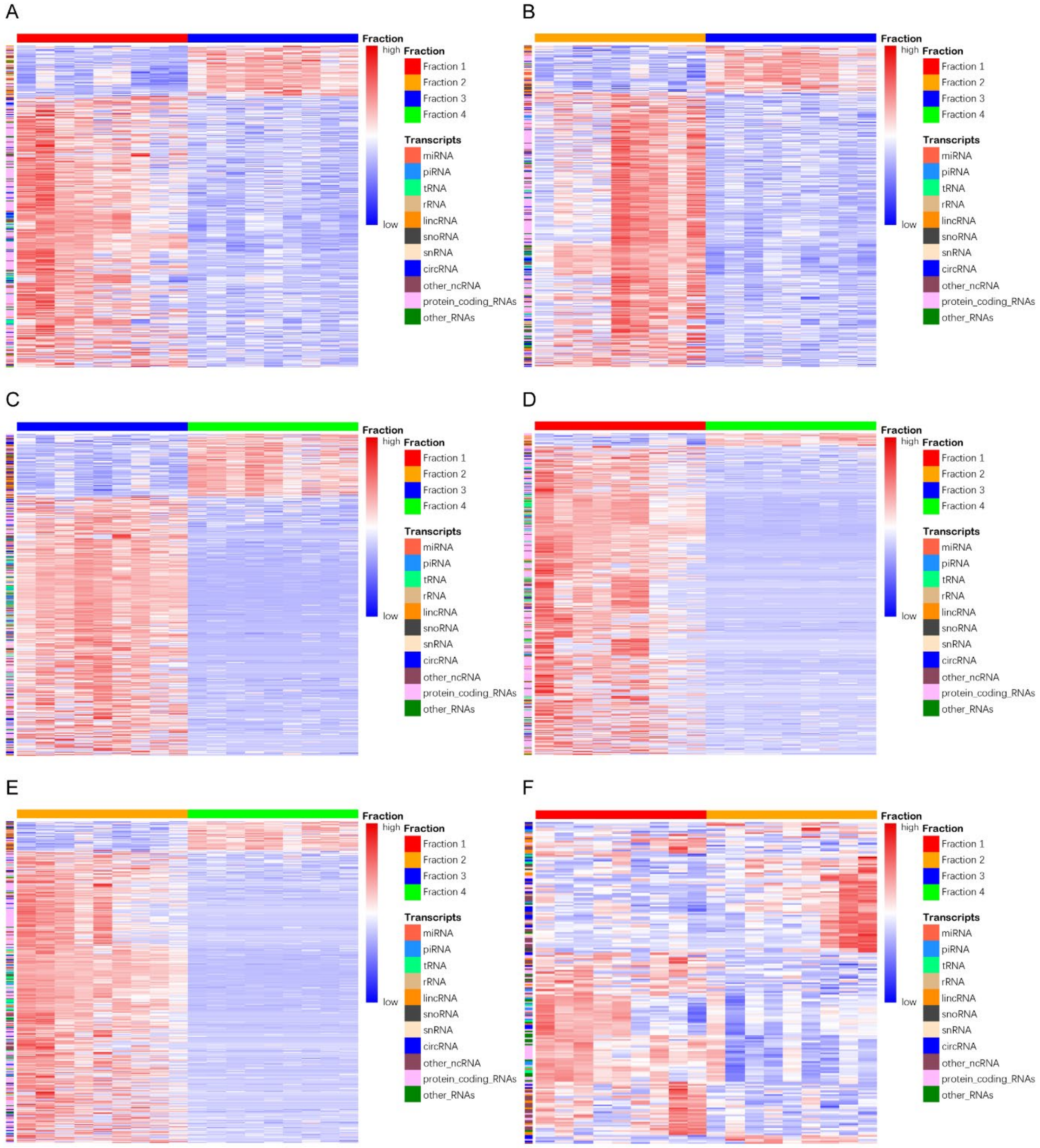


Figure S3 Heatmap showed the clustering between each two of the fractions. The fractions 1-4 represent precipitate 1, precipitate 2, EV precipitate and leftover fraction, respectively.

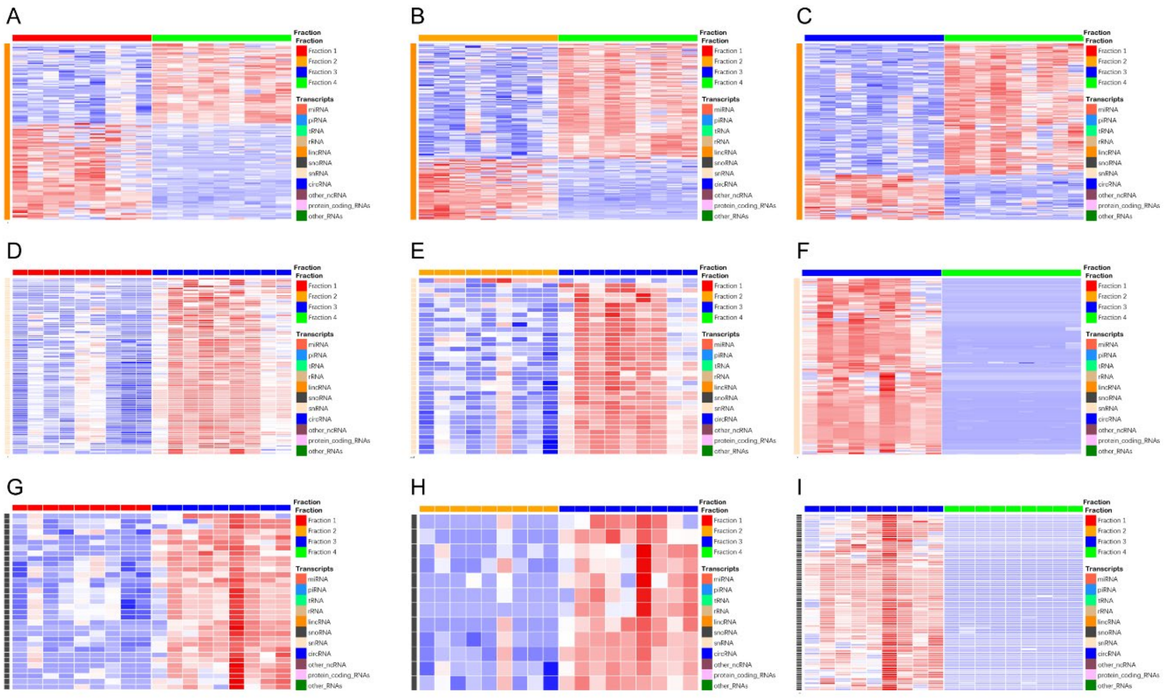


Figure S4. RNA category-specific clustering. (A-C) Heatmap based on differentially expressed lincRNAs between fraction 4 and the other 3 fractions. (D-F) Heatmap based on differentially expressed snoRNAs between fraction 3 and the other 3 fractions. (G-I) Heatmap based on differentially expressed snRNAs between fraction 3 and the other 3 fractions. The fractions 1-4 represent precipitate 1, precipitate 2, EV precipitate and leftover fraction, respectively.

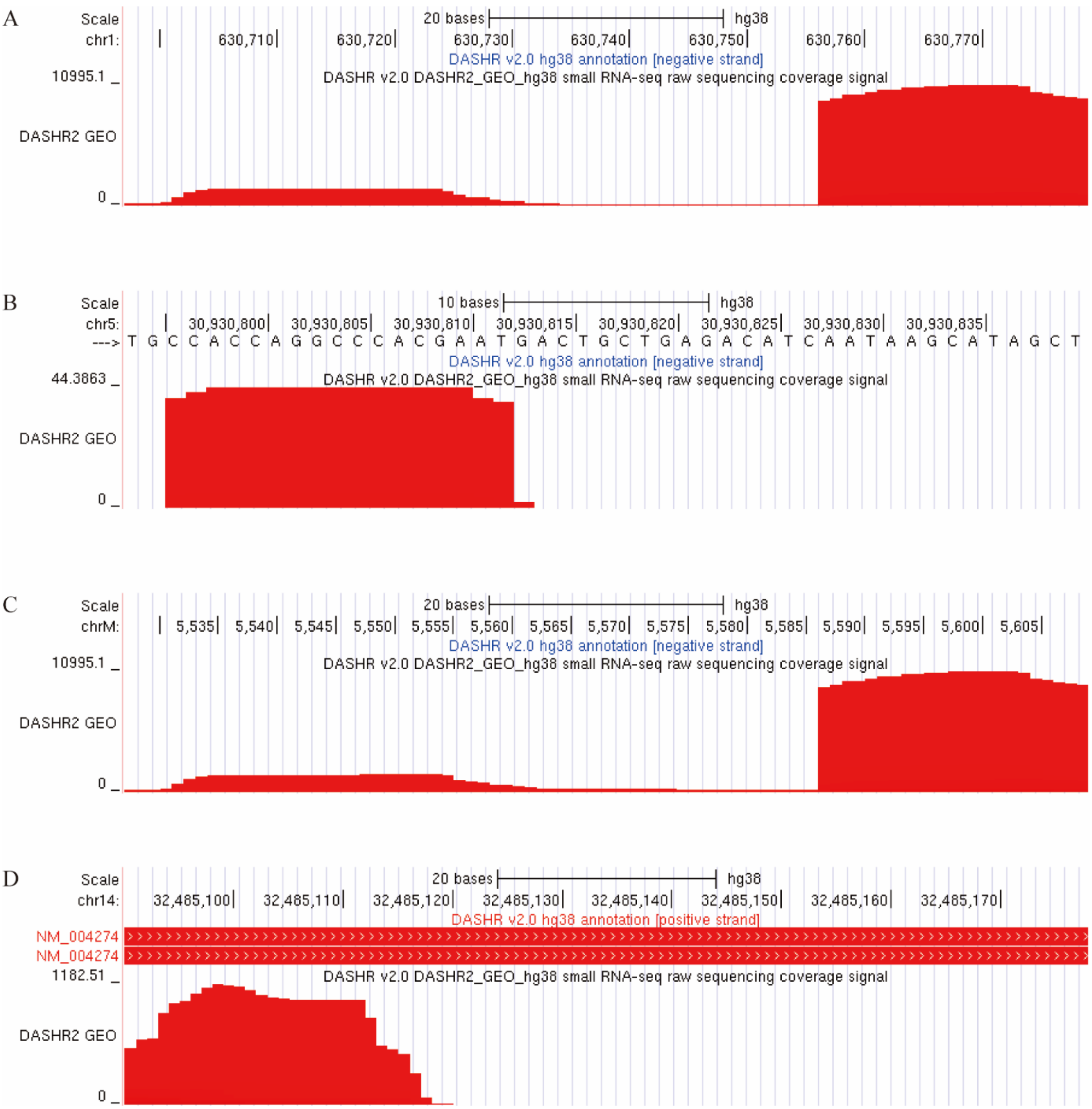


Figure S5. Representative novel miRNA loci in DASHR 2.0 database. (A) Significant peak at Chr 1: 630698-630779 (-). (B) Significant peak at chr5 30930794-30930840 (-). (C) Significant peak at chrM 5528-5609 (-). (D) Significant peak at chr14 32485091-32485178 (+).
